# Examining the role of ABC lipid transporters in pulmonary lipid homeostasis and inflammation

**DOI:** 10.1186/s12931-017-0526-9

**Published:** 2017-02-28

**Authors:** Amanda B. Chai, Alaina J. Ammit, Ingrid C. Gelissen

**Affiliations:** 1 Faculty of Pharmacy, University of Sydney, Sydney, NSW 2006 Australia; 2 Woolcock Emphysema Centre, Woolcock Institute of Medical Research, University of Sydney, Camperdown, NSW Australia; 3 School of Life Sciences, University of Technology, Sydney, NSW Australia

**Keywords:** ABC transporters, ABCA1, ABCG1, ABCA3, Lipids, Surfactant, Pulmonary inflammation

## Abstract

Respiratory diseases including asthma and chronic obstructive pulmonary disease (COPD) are characterised by excessive and persistent inflammation. Current treatments are often inadequate for symptom and disease control, and hence new therapies are warranted. Recent emerging research has implicated dyslipidaemia in pulmonary inflammation. Three ATP-binding cassette (ABC) transporters are found in the mammalian lung – ABCA1, ABCG1 and ABCA3 – that are involved in movement of cholesterol and phospholipids from lung cells. The aim of this review is to corroborate the current evidence for the role of ABC lipid transporters in pulmonary lipid homeostasis and inflammation. Here, we summarise results from murine knockout studies, human diseases associated with ABC transporter mutations, and in vitro studies. Disruption to ABC transporter activity results in lipid accumulation and elevated levels of inflammatory cytokines in lung tissue. Furthermore, these ABC-knockout mice exhibit signs of respiratory distress. ABC lipid transporters appear to have a crucial and protective role in the lung. However, our knowledge of the underlying molecular mechanisms for these benefits requires further attention. Understanding the relationship between cholesterol and inflammation in the lung, and the role that ABC transporters play in this may illuminate new pathways to target for the treatment of inflammatory lung diseases.

## Background

Asthma and chronic obstructive pulmonary disease (COPD) affect over 500 million people worldwide, contributing to significant morbidity and mortality [1, 2]. These chronic lung diseases share many features, including airway obstruction, persistent airway inflammation, and presence of multiple inflammatory mediators. However, the underlying pathophysiological processes and responses to therapy for each disease are distinct [3]. The pathogenesis of asthma involves secretion of pro-inflammatory cytokines from airway epithelial cells and macrophages [4], resulting in airway infiltration of CD4^+^ T-lymphocytes, mast cells and eosinophils. Contrastingly, CD8^+^ T-lymphocytes, macrophages and neutrophils are the more predominant cell-types found in lung tissue of patients with COPD [5]. Currently, bronchodilators including beta-agonists and anti-cholinergics, and anti-inflammatory corticosteroids are the mainstays of treatment. However, lack of disease-modifying effects of and poor responses to these therapies mean that alternate therapeutic targets and drugs are needed.

Inflammation is a complex response of the immune system to tissue injury or foreign bodies. Whilst this is considered a beneficial protective mechanism in assisting restoration of normal tissue function, persistent and excessive inflammatory responses may eventuate in disease. Recently, studies have implicated dyslipidaemia in pulmonary inflammation and various lung pathologies [6] and alongside, statins (3-hydroxymethyl-3-glutaryl coenzyme A (HMG-CoA) reductase inhibitors) have been tried as therapies for asthma and COPD with varying results [7–9].

Reverse cholesterol transport (RCT) is a protective mechanism for regulating lipid homeostasis, acting to shuttle excess cholesterol to the liver for subsequent excretion from the body. Essential to this mechanism is the interplay between ATP-binding cassette (ABC) transporters and extracellular lipid acceptors such as high-density lipoproteins (HDL) [10, 11]. Currently, 48 ABC transporters have been identified that facilitate the movement of a diverse range of substrates across cellular membranes [12]. Three ABC lipid transporters, ABCA1, ABCG1 and ABCA3, are expressed in mammalian lung cells. The first two contribute to RCT by mediating cellular cholesterol and phospholipid efflux and are transcriptionally regulated by liver X receptors (LXRs) [10, 11, 13]. ABCA3, a phospholipid exporter, is specifically involved in pulmonary surfactant production. Surfactants in the lung contain approximately 90% phospholipids and 10% surfactant proteins and are essential to prevent alveolar collapse by reducing surface tension at the air-liquid interface [14, 15]. Studies in lungs of knockout mice have established that absence of ABC transporters result in disrupted lipid homeostasis, diminished surfactant production, impaired respiratory physiology and increased expression of inflammatory cytokines in lung cells [10, 15, 16]. Furthermore, mutations to these transporter genes are associated with human lung diseases such as alveolar proteinosis and respiratory distress syndrome [14, 17, 18]. Given that hyperlipidaemia has been demonstrated to accelerate inflammation in murine bronchoalveolar lavages [19], the role of ABC transporters in the pathogenesis of inflammatory lung diseases is an exciting and promising avenue to explore.

This review will explore current available knowledge regarding the roles of ABCA1, ABCG1 and ABCA3 in maintaining lipid homeostasis and controlling inflammation in the lung. Clarifying this relationship between pulmonary lipidosis and inflammation will help guide relevant future research studies that could ultimately identify an alternative therapeutic angle for the treatment of asthma, COPD and other related conditions.

## ABCA1

ABCA1 is widely expressed throughout the body and contributes to RCT by exporting cholesterol and phospholipids out of cells to extracellular acceptors [20, 21]. In the periphery, the lipid-poor HDL protein component apolipoprotein (apo)A-I, is the predominant extracellular acceptor, and binding of apoA-I to cells expressing ABCA1 leads to the generation of small nascent HDL particles [22, 23]. Homozygous and heterozygous mutations to the *ABCA1* alleles result in Tangier disease and familial hypoalphalipoproteinaemia respectively, with only around 100 Tangier patients diagnosed worldwide [24]. Phenotypically, these patients have markedly reduced HDL levels, resulting in lipid accumulation in numerous tissues and subsequent increased risks of atherosclerosis and cardiovascular complications [25]. Furthermore, it was recently shown that ABCA1-mutation carriers exhibit signs of systemic inflammation as indicated by elevated levels of circulating inflammatory cytokines [24]. Over 73 mutations, including single nucleotide polymorphisms have also been reported in the *ABCA1* gene [26], raising the possibility of more widespread cholesterol metabolic disruptions across populations. The contributions of such mutations to development of asthma and COPD are yet to be explored.

**Fig. 1:**
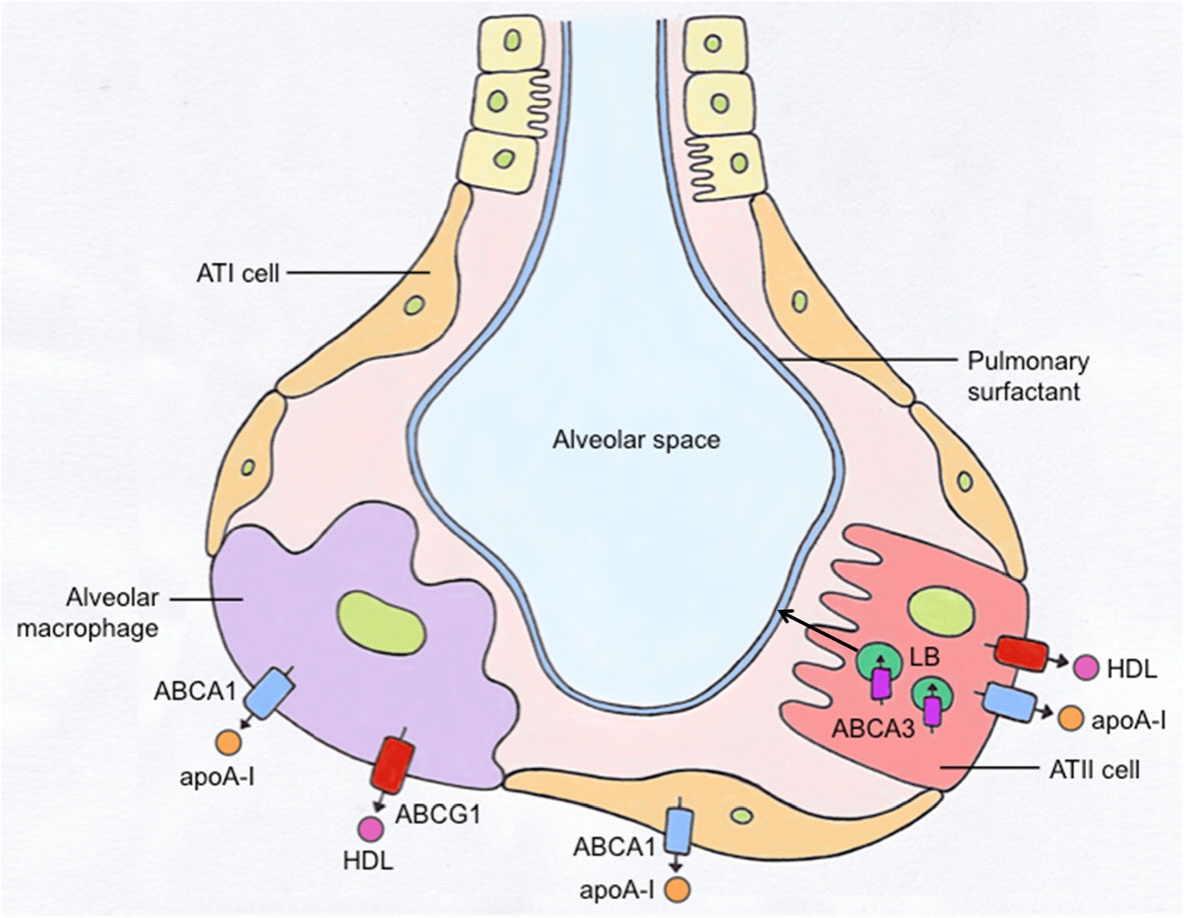
ABC transporters expressed in various cell types of the alveolus. Three ABC transporters, namely ABCA1, ABCG1 and ABCA3, are expressed in lung cells present in the alveolus, notably the alveolar epithelial type I and II cells (ATI and ATII respectively) that line the alveoli or air sacs, and alveolar macrophages that are phagocytes of the pulmonary immune system. LB – lamellar bodies, where ABCA3 contributes lipids that eventually are secreted as surfactants (*represented* via *the black arrow*)

ABCA1 tissue expression is second highest in the lungs, after the liver, suggesting a critical role for the transporter and lipid homeostasis in pulmonary function [27]. Immunohistochemical data have identified the presence of ABCA1 in alveolar type I (ATI) and type II (ATII) pneumocytes and alveolar macrophages [28, 29] (see Table 1 and Fig. 1). Cholesterol loading and treatment with LXR-agonists, both separately and conjointly, were also able to induce ABCA1 expression and function in human airway smooth muscle (ASM) cells [30].

**Table 1:** Cellular expression and substrates of ABC transporters expressed in mammalian lungs

Transporter	Expressed in which lung cells	Reported substrates	References
ABCA1	Alveolar macrophages, airway smooth muscle cells, type I pneumocytes, type II pneumocytes	Cholesterol, phosphatidylcholine, phosphatidylserine, sphingolipid 1-phosphate, sphingomyelin	[28–30, 83]
ABCG1	Alveolar macrophages, airway smooth muscle cells, type II pneumocytes, T-lymphocytes, epithelial cells, dendritic cells	Cholesterol, oxysterols, phosphatidylcholine, sphingomyelin	[43, 47, 49, 84]
ABCA3	Type II pneumocytes	Cholesterol, phosphatidylcholine, phosphatidylglycerol, sphingomyelin	[15, 60, 62, 63]

### Phenotype of ABCA1-knockout mouse models

The critical role of ABCA1 in lung inflammation is evident from murine knockout models. *Abca1*-knockout mice exhibit interrupted lipid export, and a 70% reduction in plasma cholesterol and phospholipids due to virtually undetectable HDL and apoA-I levels, compared to wild-type [10]. Phenotypically, these mice at 4 months of age exhibit massive cholesterol and phospholipid accumulation in alveolar macrophages and ATII pneumocytes, as compared with wild-type littermates [10]. Furthermore, oil red-O staining of *Abca1* ^*−/−*^ lungs revealed pulmonary focal lesions, and microscopic analysis revealed enlarged foamy alveolar macrophages and ATII cell hyperplasia, indicating alveolar proteinosis [10]. Physiologically, these mice experienced reduced tidal volume and hyperventilation, although it was unclear whether this respiratory distress was directly attributable to abnormalities in the lung or in other organs [10]. Nevertheless, these findings, coupled with similar but progressively severe observations in 7-, 12- and 18-month-old mice [31], suggest that absence of ABCA1 results in age-dependent progressive pulmonary disease. Supporting the importance of this ABCA1-mediated pathway in normal lung physiology is the finding that apoA-I^−/−^ mice have elevated inflammatory cell infiltration (particularly neutrophils and leukocytes), collagen deposition and airway hyper-responsiveness, and impaired pulmonary vasodilatation [32]. These observations strongly implicate a role of ABCA1-mediated RCT in inflammatory lung diseases.

### Mechanisms of ABCA1-mediated RCT and role in pulmonary inflammation

The mechanism by which ABCA1 exports lipids has been predominantly studied in the context of its role in macrophage lipid homeostasis and the development of atherosclerosis. ABCA1 is thought to export lipids from the inner to the outer leaflet of the cell membrane via an ATP-dependent mechanism [12]. The subsequent method of transfer of these lipids to apoA-I is still open for debate. One model proposes that hydrolysis of ATP causes a conformational change in the transporter, enabling apoA-I to bind [33]; alternatively, apoA-I may bind and solubilise lipids in exovesiculated membrane lipid domains that arise from membrane asymmetry [34]. It is worth noting that evidence for the presence of apoA-I in the lung is currently limited. Bates and colleagues [28] were able to stain cryosections of murine lungs with anti-mouse apoA-I antibody, to verify that adequate levels of apoA-I exist for interaction with pneumocyte ABCA1. Meanwhile, Delvecchio and colleagues [30] performed in vitro experiments involving incubation of ASM cells with *exogenous* apoA-I, and were able to confirm the capacity of ASM ABCA1 to efflux lipids to apoA-I. Of further interest was the finding that apoA-I is reduced in sputum samples of patients with COPD compared to controls (which were smokers), with the authors suggesting that apoA-I could potentially be investigated as a biomarker [35]. Inhaled apoA-I mimetic peptides, which are under development as treatments for atherosclerosis, have been suggested as a potential treatment for inflammatory lung conditions [36].

Studies addressing the mechanisms by which ABCA1 activity affects inflammatory processes in the lung have investigated various pathways. Firstly, it has been proposed that excess cholesterol itself is pro-inflammatory and acts as the trigger for the cellular inflammatory response [37], and hence ABCA1 activity may simply be anti-inflammatory by removing excess cholesterol. Alternatively, other studies have investigated its transcriptional upregulation via LXR. In human alveolar macrophages, LXR-agonists that increased ABCA1 mRNA expression were shown to reduce LPS-induced airway inflammation and neutrophil production. However, explanations for the underlying mechanisms are conflicting. In one study, this was attributed to a NF-κB-dependent pathway [38], whereas another study found no involvement of either of the pro-inflammatory transcription factors NF-κB or AP-1 [29]. However, it is worthwhile noting that LXR also affects ABCG1 expression (covered in the next section), effects on which were not accounted for in the latter study.

Lastly, Dai and colleagues have investigated effects of ABCA1 overexpression in alveolar macrophages and showed that the anti-inflammatory effects of ABCA1 may occur via suppression of granulocyte-colony stimulating factor, which subsequently could reduce the involvement of inflammatory cells such as neutrophils [39]. Evidently, further research is required to clarify the mechanisms by which ABCA1 activity affects inflammatory pathways.

### ABCA1 and asthma

The association between ABCA1 function and asthma is still a novel and underdeveloped area. Mice overexpressing human ABCA1 exhibited reduced neutrophil count, IgE levels, peri-bronchial inflammation and airway epithelial thickness in response to daily ovalbumin challenges when compared to wildtype mice [39]. Furthermore, treatment of house dust mite-challenged mice with intranasal apoA-I was able to minimise eosinophilia, neutrophilia, interleukin (IL)-17E, IL-33 and airway hyperresponsiveness [40]. The same authors also revealed that apoA-I levels in bronchoalveolar lavage fluid of asthmatic patients were significantly lower than that from healthy subjects [40]. These findings suggest that upregulation of ABCA1 and/or apoA-I activity could be beneficial in patients with inflammatory lung diseases.

In summary, ABCA1 plays crucial roles in the maintenance of lipid homeostasis and modulation of inflammatory responses in lung cells. However, further studies are necessary to elucidate the exact mechanisms by which these effects occur and whether therapeutic targeting of these pathways will be effective.

## ABCG1

Like ABCA1, ABCG1 is also ubiquitously expressed in the body [41]. ABCG1 is considered a half-transporter and requires dimerization to be functional, unlike ABCA1 which is a full-transporter that functions independently [42]. Whereas ABCA1 contributes to formation of nascent HDL particles, ABCG1 is thought to export lipids to larger, more mature HDL subclasses [43]. It has been suggested from cell culture as well as murine knockout models that ABCA1 and ABCG1 may synergise together [42, 44].

ABCG1 has also mostly been studied in the context of atherosclerosis, however there are currently no reported genetic ABCG1 mutations reported that are associated with an increased risk for cardiovascular disease in humans. Interestingly, ABCG1 deficiency in human alveolar macrophages has been associated with pulmonary alveolar proteinosis (PAP), a rare condition characterised by accumulation of surfactant proteins and lipids in the alveolar space [18]. This will be discussed in detail below.

Similarly to ABCA1, ABCG1 utilises ATP to actively export its substrates (see Table 1 and Fig. 1) to the exoplasmic leaflet of the cell membrane [42]. However, direct binding of ABCG1 with an acceptor (e.g. HDL) is not required for transport activity, and there is currently some debate in the literature whether the transporter is exclusively located intracellularly or travels and acts on the cell surface [45].

### ABCG1 and its role in lipid homeostasis and pulmonary inflammation

There is strong evidence derived from *Abcg1*-knockout murine models to suggest that this transporter also plays an essential role in maintaining pulmonary lipid homeostasis. ABCG1 is widely expressed in various lung cell types, including alveolar macrophages, epithelial cells, ATII pneumocytes, ASM cells, T-lymphocytes and dendritic cells [43, 46, 47]. Several studies in *Abcg1*-knockout mice have demonstrated disrupted lipid homeostasis in murine lungs. Compared to heterozygote *Abcg1* ^*+/−*^ mice, homozygote knockouts revealed massive accumulation of lipids in the alveolar macrophages. The subpleural region of *Abcg1* ^*−/−*^ lungs also revealed build-up of lymphocytes, multinucleated giant cells, and macrophage foam cells containing an abundance of cholesterol clefts that indicated impaired lipid processing. These observations were progressive and age-dependent, and were exacerbated upon administration of a high-fat high-cholesterol diet [16, 43]. Furthermore, human *ABCG1*-transgenic mice overexpressing the ABCG1 transporter were protected from this diet-induced pulmonary lipidosis [43].

Absence of ABCG1 also results in progressive and chronic pulmonary inflammation [19]. Immunofluorescence studies and bronchoalveolar lavages obtained from chow-fed *Abcg1* ^*−/−*^ mice revealed multiple markers of inflammation, including elevated levels of foamy macrophages, lymphocytes and pro-inflammatory cytokines. None of these features were observed in lungs of wild-type littermates [19]. Coupled with the observations that administration of a high-fat high-cholesterol diet significantly accelerated lipid deposition and induced macrophage cytokine expression in wild-type and (to an even greater extent in) *Abcg1* ^*−/−*^ lungs, these findings imply that ABCG1 indirectly controls inflammation by modifying intracellular cholesterol levels [19]. However, the timeline of events was less clear in a separate study, which also reported inflammatory cytokine, neutrophil and lymphocyte infiltration alongside lipidosis in the lungs of *Abcg1* ^*−/−*^ mice [48]. Wojcik and coauthors proposed that one of two mechanisms could be occurring: i) lipid accumulation causes inflammation or ii) absence of *Abcg1* triggers inflammation that subsequently leads to the observed lipidosis [48]; which one of these events predominates is still unclear. Nevertheless, pulmonary ABCG1 expression also appears to protect against exogenous inflammation-inducing factors. *Abcg1* ^*−/−*^ mice challenged with LPS or gram-negative bacteria displayed an exaggerated pulmonary response, characterised by elevated neutrophil, leukocyte, cytokine and chemokine levels in the airspace and tissue remodelling as compared to wild-type mice [49]. These effects were attributed specifically to disrupted *Abcg1* ^*−/−*^ activity in alveolar macrophages. Despite the enhanced pulmonary bacterial clearance in these knockout mice, the excessive inflammation meant they also had a much higher mortality rate [49]. In a murine model of allergic asthma, ovalbumin-challenged *Abcg1* ^*−/−*^ mice displayed increased airway neutrophils and IL-17, which are implicated in severe forms of asthma [46]. These findings suggest that ABCG1 plays a critical role in regulating the host defence response in the lung. Overall, results from murine models provide strong evidence for the critical role of ABCG1 in maintaining cellular lipid homeostasis and controlling pulmonary inflammation.

### Inflammatory profile of ABCG1-knockout mice

Amongst the myriad of inflammatory cytokines and chemokines released in asthma and COPD, several have also been identified in *Abcg1*-knockout mice. Levels of the inflammatory cytokines tumour necrosis factor (TNF)-α and IL-1β were markedly elevated in lungs of *Abcg1*-deficient chow-fed mice [19]. TNF-α is expressed in multiple lung cell types, including macrophages, lymphocytes, mast cells and ASM cells, and promotes oxidative damage that subsequently activates NF-κB and AP-1. Results of this activation include recruitment of adhesion molecules, lymphocytes, and other inflammatory cytokines, and increased airway hyperresponsiveness [50]. IL-1β has been shown to cause inflammation, airway fibrosis, bronchiolar thickening and mucus metaplasia – features that are prominent in COPD and asthma. Furthermore, IL-1β enhanced production of matrix metalloproteinases (MMP)-9 and −12 in neutrophils and macrophages of murine airways respectively [51]. MMPs that degrade extracellular matrix are overexpressed in patients with COPD and asthma, and have been associated with airway inflammation and remodelling [52]. Elevated levels of MMP-8 and MMP-12 have also been reported in lungs of *Abcg1* ^*−/−*^ mice [19]. Alveolar macrophages also play a pivotal role in orchestrating the inflammatory processes in COPD and asthma by secreting numerous pro-inflammatory cytokines and chemokines [3]. However, they also secrete inhibitory mediators such as IL-10 that dampen inflammation, but this secretion is thought to be impaired in patients with asthma [53]. These activities were reflected in the alveolar macrophages of *Abcg1* ^*−/−*^ mice, with Wojtik and coauthors reporting significantly elevated pro-inflammatory IL-1, IL-6 and IL-12 levels, and decreased expression of the anti-inflammatory cytokine IL-10 [48]. Another study indicated that following an asthma allergen challenge in *Abcg1* ^*−/−*^ mice, airway neutrophil and IL-17 levels were elevated compared to wild-type [46]. Although it was not specified which IL-17 subtype was elevated, a separate study showed that IL-17A contributes to glucocorticoid-insensitivity by decreasing the activity of histone deacetylase-2, an enzyme that normally mediates the anti-inflammatory effects of glucocorticoids [54]. Taken together, evidence from *Abcg1* ^*−/−*^ mouse models suggest that disruption to ABCG1 activity results in a phenotype reflective of inflammatory lung disease.

### ABCG1 and pulmonary surfactant homeostasis

ABCG1 deficiency has been identified in patients with pulmonary alveolar proteinosis (PAP), a condition characterised by pulmonary surfactant accumulation [18]. Pulmonary surfactant is produced in lamellar bodies of ATII pneumocytes, and degraded by alveolar macrophages. Studies in chow-fed *Abcg1* ^*−/−*^ mice showed accumulation of ATII pneumocytes that were enlarged and engorged with surfactant-rich lamellar bodies, suggesting abnormal surfactant clearance. Esterified cholesterol levels were also elevated in alveolar macrophages, which occurred despite increased *Abca1* expression, which was thought to be due to compensatory upregulation. The authors hypothesised that ABCG1 disruption in both these cell types resulted in defective lipid/surfactant secretion, thereby resulting in compensatory cell hypertrophy and severe pulmonary lipidosis [16]. In 90% of PAP cases, autoantibodies that neutralise granulocyte macrophage-colony stimulating factor (GM-CSF) impair alveolar macrophage maturation, consequently hindering their ability to clear surfactant [55]. Thomassen and colleagues recognised that both PAP patients and GM-CSF-knockout mice demonstrate surfactant accumulation in alveolar macrophages, and ABCG1 deficiency, despite increased ABCA1 activity [18]. Induction of ABCG1 expression via LXRα was able to reverse intracellular lipid accumulation and restore lung compliance in GM-CSF-knockout mice [56, 57]. These results demonstrate that ABCG1 is critical for normal surfactant metabolism.

Altogether, the importance of ABCG1 in regulating lipid levels and inflammation in the lung is clear from murine studies, as well as the phenotype of patients with PAP. However, the underlying mechanisms and pathways by which ABCG1 mediates its protective effects are currently unclear.

## ABCA3

Unlike ABCA1 and ABCG1 that are expressed in multiple lung cell types, ABCA3 is exclusively expressed in the lamellar bodies of ATII pneumocytes [58] and crucial for the proper formation of lamellar bodies in these cells [59]. Due to its intracellular expression, ABCA3 does not require an extracellular lipid acceptor. Substrates of ABCA3 include phosphatidylcholine, phosphatidylglycerol and also cholesterol (see Table 1 and Fig. 1), which are transported into the lamellar bodies [60, 61]. Within these organelles, phospholipids are combined with surfactant proteins to produce pulmonary surfactant, which is subsequently exocytosed into the alveolar airspace to reduce surface tension and prevent alveolar collapse [15, 62]. The exact mechanism of ABCA3-mediated lipid transport remains elusive [63].

### ABCA3 and its role in lipid homeostasis and pulmonary inflammation

Dysfunctional ABCA3 disrupts lamellar body phospholipid export, causing respiratory distress syndrome (RDS), and pediatric and adult interstitial lung disease [14, 60], which is a family of related conditions characterised by inflammation and fibrosis of the pulmonary interstitium [60]. RDS typically affects neonates, and is caused by surfactant deficiency that results in alveolar collapse, hypoxaemia and pulmonary oedema [14], hence this condition is often lethal. Inflammation accompanying RDS is fuelled by neutrophils and cytokines that are likely controlled via the NF-κB pro-inflammatory signalling pathway [64]. Corticosteroids can be used for their ability to stimulate surfactant phospholipid synthesis in surviving infants, but can have significant side effects. These compounds have also been found to upregulate ABCA3 expression and modulate transcription factors including NF-κB in chronic inflammatory lung diseases [58, 65]; these may be additional mechanisms by which they facilitate foetal lung maturation.

Several murine knockout studies have also confirmed the critical role for ABCA3 in surfactant production. Absence of mature lamellar bodies and surfactant phospholipids in the alveolar space of *Abca3* ^*−/−*^ mice resulted in surfactant deficiency and fatal respiratory failure [15, 61]. One proposed mechanism for the disrupted surfactant production seen in RDS suggests that ABCA3 mutations elevate endoplasmic reticulum stress and trigger apoptosis of ATII cells [62]. Elaborating on this finding, a more recent study showed that ABCA3 expression protects ATII cells from free cholesterol-induced cytotoxicity by exporting free cholesterol. Cells transfected with a non-functional mutant of ABCA3, ABCA3-Q215K, displayed excessive accumulation of cholesteryl esters and total phospholipids. The study showed that the excess lipids in these cells were rerouted away from lamellar bodies towards the ER, where the subsequent lipid accumulation resulted in endoplasmic reticulum stress and thus ATII apoptosis [60]. There is also increasing evidence that ATII cell dysfunction and surfactant abnormalities are implicated in the pathogenesis of COPD. Surfactant dysfunction is thought to contribute to airway resistance, oxidative stress, inflammation and increased protease activity that promotes tissue remodelling [66]. A mouse model expressing a common ABCA3 mutation observed in patients with diffuse parenchymal lung disease, the ABCA3^E292V^ mutation, reported spontaneous lung remodelling in these mice with an emphysema-like phenotype [67]. However, no clinically significant ABCA3 mutations have been identified in COPD patients as yet [68].

Evidence is currently limited with respect to a potential role for ABCA3 in inflammation, despite suggestions that it may be involved in transport of cholesterol. Bronchoalveolar lavage cells from lungs of *Abca3* ^*−/−*^ mice showed no statistically significant increase in expression of inflammatory cytokines, although measurements were only made for five cytokines [69]. In patients with ABCA3 mutations, levels of the representative cytokine IL-8 were significantly elevated in ATII cells as compared to normal cells [17]. It is unknown whether the increase in IL-8 was due to lipid accumulation within the ATII cells.

An alternate mechanism by which ABCA3-deficiency may lead to inflammation may be due to the associated reduction in the secretion of surfactant proteins. The hydrophilic collectins SP-A and SP-D have immunomodulatory functions, and have been suggested to reduce inflammation in the lungs by enhancing phagocytosis of apoptotic cells and pathogens, and inhibiting T-cell function. A deficiency in their secretion has been associated with the pathogenesis of COPD and asthma, although previous attempts at administering surfactants to asthma patients had mixed results [70–72]. Chiba and colleagues also demonstrated that both SP-A and phosphatidylglycerol in surfactant have anti-inflammatory effects [72]. Since it is clear that ABCA3 is critical for lamellar body formation, it is possible that ABCA3 indirectly modulates lung inflammation by helping to form the organelles that are necessary to produce these anti-inflammatory surfactant components.

In summary, our understanding of a role for ABCA3 in regulating lung lipid levels and inflammation is not as expansive as that for ABCA1 and ABCG1. Its role in the lung has been well defined in the context of surfactant production, however how it modulates pulmonary inflammation warrants further investigation.

### Statins as alternate therapy for inflammatory lung disease

Recently, attention has been given to statins to explore their effect on lung inflammation in asthma and COPD. In addition to their lipid-lowering effects, statins have pleiotropic anti-inflammatory, antioxidant and anti-proliferative properties. In vivo studies in rodents have been promising, with simvastatin attenuating tobacco-induced infiltration of macrophages, neutrophils and leukocytes into the airways [9], and reducing the expression of macrophages, neutrophils, eosinophils, MMPs and various inflammatory cytokines in bronchoalveolar lavage fluid in ovalbumin-induced allergic asthma [73]. However, the benefits of statins in humans with inflammatory lung conditions have so far been inconsistent. Observational studies in humans have shown that statins can reduce exacerbation and mortality rates, and improve symptom control in patients with asthma and COPD [8, 74, 75]. Furthermore, a randomised placebo-controlled study in 12 COPD patients reported significantly reduced levels of inflammatory cells and mediators in the lung following atorvastatin treatment, although this did not translate into improved lung function [76]. On the other hand, one randomised double-blind crossover trial found no anti-inflammatory activity in asthma patients given simvastatin [77], while a large-scale randomised placebo-controlled trial in 884 patients concluded that simvastatin was ineffective in reducing exacerbations in moderate-to-severe COPD [78]. Two systematic reviews have reported statistically insignificant improvements in symptom control or lung function in asthma patients, however data did support statin-associated reductions in airway inflammation [79, 80]. A possible explanation for the inconsistent effects of statins may be their negative effects on ABC transporter expression. It has been well established that statin treatment in cells negatively affect ABCA1 and ABCG1 expression via an LXR dependent mechanism [81, 82]. This may have offset some of the benefits associated with their cholesterol-lowering and pleiotropic anti-inflammatory effects.

## Conclusions

Although much of our understanding of the function of ABC lipid transporters has been derived from studies in the context of cardiovascular disease, there has been increasing interest in their activities in the lung. Studies utilising murine knockout models have reported congruent results that support the crucial roles of ABCA1 and ABCG1 in maintaining lipid homeostasis in alveolar macrophages, ASM cells, and ATI and ATII pneumocytes. Furthermore, many studies have affirmed a role of ABCG1 in protecting against lung inflammation, whereas positive, albeit less evidence is available for ABCA1, and thus this is an area requiring further investigation. Human pathologies such as RDS demonstrate a clear link between ABCA3 dysfunction and lipid disruption in ATII cells. Although a direct relationship between ABCA3 and inflammation has yet to be established, currently evidence suggests it plays an indirect role via the secretion of surfactant proteins. Statins have also shown mixed clinical benefits in improving outcomes in asthma and COPD, and any positive effects have been attributed to their pleiotropic anti-inflammatory activity. Other potential therapies such as inhaled apoA-I mimetic peptides have been suggested but so far not tested. Overall, ABC transporters are a promising area to further explore in the search for more effective therapies for inflammatory lung diseases.

## Abbreviations

ABC: ATP-binding cassette
AP-1: Activator protein-1
apo: Apolipoprotein
ASM: Airway smooth muscle
ATI: Alveolar type I
ATII: Alveolar type II
ATP: Adenosine triphosphate
COPD: Chronic obstructive pulmonary disease
GM-CSF: Granulocyte macrophage-colony stimulating factor
HDL: High-density lipoprotein
HMG-CoA: 3-hydroxymethyl-3-glutaryl coenzyme A
IL: Interleukin
LPS: Lipopolysaccharide
LXR: Liver X receptor
MMP: Matrix metalloproteinase
NF-κB: Nuclear factor κB
PAP: Pulmonary alveolar proteinosis
RCT: Reverse cholesterol transport
RDS: Respiratory distress syndrome
SP: Surfactant protein
TNF-α: Tumour necrosis factor-α
